# A PPG Signal Dataset Collected in Semi-Naturalistic Settings Using Galaxy Watch

**DOI:** 10.1038/s41597-025-05152-z

**Published:** 2025-05-28

**Authors:** Sangjun Park, Dejiang Zheng, Uichin Lee

**Affiliations:** Korea Advanced Institute of Science and Technology, School of Computing, Daejeon, 34141 South Korea

**Keywords:** Medical research, Biomarkers, Population screening, Prognosis

## Abstract

The widespread adoption of consumer-grade wearable devices, such as Galaxy Watch, has revolutionized personal health monitoring as they enable continuous and non-invasive measurement of key cardiovascular indicators through photoplethysmography (PPG) sensors. However, existing datasets primarily rely on research-grade devices, limiting the applicability of consumer-grade wearables in real-world conditions. To address this gap, this study presents GalaxyPPG, a dataset collected from 24 participants that includes wrist-worn PPG signals from a Galaxy Watch 5 and an Empatica E4, alongside chest-worn ECG data from a Polar H10. Data were captured during diverse activities in a semi-naturalistic setting, providing insights into the sensing performance of consumer-grade wearables under motion- or stress-inducing activities. This dataset is designed to advance applications of PPG signals, such as HR tracking with diverse physical activities and HRV monitoring for stress detection. Additionally, we offer an open-source toolkit for data collection and analysis using Samsung Galaxy Watch, fostering reproducibility and further research leveraging this toolkit.

## Background & Summary

The emergence and advancement of consumer-grade wearable devices such as smartwatches (e.g., Galaxy Watch, Apple Watch) have dramatically transformed personal health monitoring methods. In particular, photoplethysmography (PPG) sensors embedded in smartwatches can continuously and non-invasively measure key cardiovascular indicators such as heart rate (HR) and heart rate variability (HRV), providing a valuable foundation for cardiovascular health management^1^. Moreover, the versatility of PPG signals enables the extraction of various physiological parameters, including oxygen saturation and respiratory rate. PPG sensing offers a substantial potential for broad digital healthcare applications, including sleep monitoring^2^ and stress assessment^3^. This development has prompted clinical and research settings to actively explore a wide range of opportunities for leveraging consumer-grade wearable devices.

However, despite this potential, using PPG signals in everyday conditions faces a significant challenge from motion artifacts (MA)^4^. MAs occur when physical movements, such as walking, running, or changing wrist positions, alter the contact between the sensor and the skin and the movement of surrounding tissues, introducing irregular noise into the signal^5^. This complicates the stable extraction of key physiological indicators, such as heart rate, and can erode the inherent advantage of continuous monitoring that PPG provides. However, consumer-grade wearable devices, such as Galaxy Watch, have not been systematically validated to assess how MAs occur and affect data quality, often hindering clinical and research applications. Addressing this issue requires sustained research and verification efforts, including improvements in signal processing algorithms, noise reduction techniques, and data preprocessing strategies.

In the past, researchers introduced various datasets to address challenges like MAs and explore diverse PPG signal analysis applications. The IEEE Signal Processing Cup dataset^6^ focuses on accurate heart rate (HR) measurement in environments with intense physical activities, such as walking and running, where MAs are prevalent. PPG-DaLiA^5^, on the other hand, captures daily activities in semi-naturalistic environments, primarily focusing on analyzing motion artifacts. WESAD^3^ focuses on physiological and behavioral sensing under stress-inducing stationary activities, such as interviewing. However, these datasets are tailored to their specific purposes and primarily rely on research-grade devices like the E4 wearable or custom-built PPG sensors for data collection. As a result, the applicability of consumer-grade wearable devices has not been adequately addressed.

Building on these considerations, our dataset integrates multiple sensing modalities to enable robust validation of PPG signals in semi-naturalistic settings. Specifically, it comprises simultaneously collected data from 24 participants wearing three devices: a Polar H10 chest-worn electrocardiogram (ECG) sensor, a wrist-worn Empatica E4 device commonly used in research contexts for PPG measurements, and a smartwatch, the Galaxy Watch 5. Data collection involved various everyday activities designed to introduce motion artifacts and a stress-inducing test to demonstrate potential applicability in stress detection and related domains. The Galaxy Watch was chosen because it officially supports PPG data export via Samsung Health Sensor Software Development Kit (SDK), making it a suitable platform for PPG sensor data collection and model evaluation. This collection setup allows for comparing and assessing signal quality across different devices and conditions, ultimately contributing to a clearer understanding of PPG performance in real-world environments. Additionally, we have released research toolkits for Galaxy Watch data collection and analysis to facilitate similar experiments and enable other researchers to reproduce and extend this experimental framework (described in the Code Availability Section).

## Methods

### Physiological Sensor Data Toolkit for Galaxy Watch

**Fig. 1:**
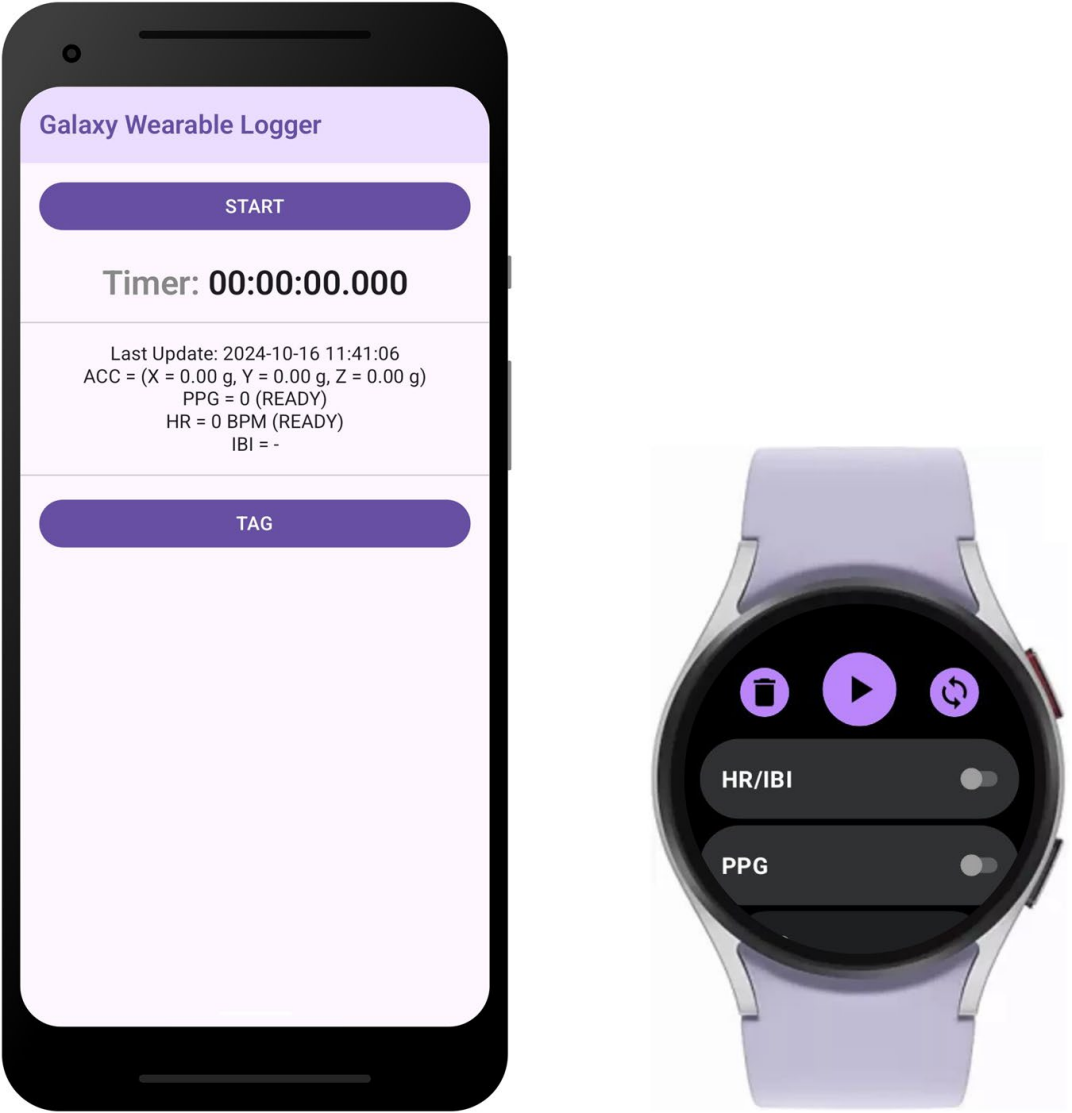
Interface of the Galaxy Wearable Logger toolkit: The smartphone app (left) and Galaxy Watch app (right) facilitate synchronized data collection, including PPG, heart rate, and acceleration signals, enabling seamless logging for research and analysis.

The authors developed a custom data acquisition toolkit to collect raw sensor data using the Galaxy Watch. The toolkit was implemented through the *Samsung Health Sensor SDK*^7^ (referred to as Samsung Privileged SDK at the time), officially provided by Samsung for assessing signals such as accelerometer, PPG, HR, and skin temperature. The implemented application consisted of two separate apps: one for the Galaxy Watch and another for the Android smartphone. The Galaxy Watch app allows users to start or stop data collection by selecting the data to be collected from HR/IBI, PPG, ACC, and skin temperature. The smartphone application was designed to monitor the data collection in real time. To support this, it included features to display the updated status of each sensor’s data, as shown in Fig. 1. Further, a timer was implemented to help researchers track elapsed time, and a tagging button was included to log timestamps for the activity transition during the experiment. After the experiment, the collected data could be exported and downloaded on the smartphone app as a zip file containing multiple CSV files.

### Data Collection

#### Ethics Approval

Our study for building the GalaxyPPG dataset was approved by the Institutional Review Board (IRB) of the Korea Advanced Institute of Science and Technology (KH2024-109). Prior to data collection, all participants were informed about the purpose and procedures of the study, and provided written informed consent for both participation and data sharing.

#### Recruitment

We recruited participants through our campus’s online bulletin board. Participants are restricted to right-handed, physically healthy adults aged 18–65, especially those with no history of heart conditions or cardiovascular issues. Based on the recruitment, 24 participants (12 females) with a mean age of 23.3 (SD = 2.0; range = 20-29) participated in our experiment.

#### Collection Setup

**Fig. 2:**
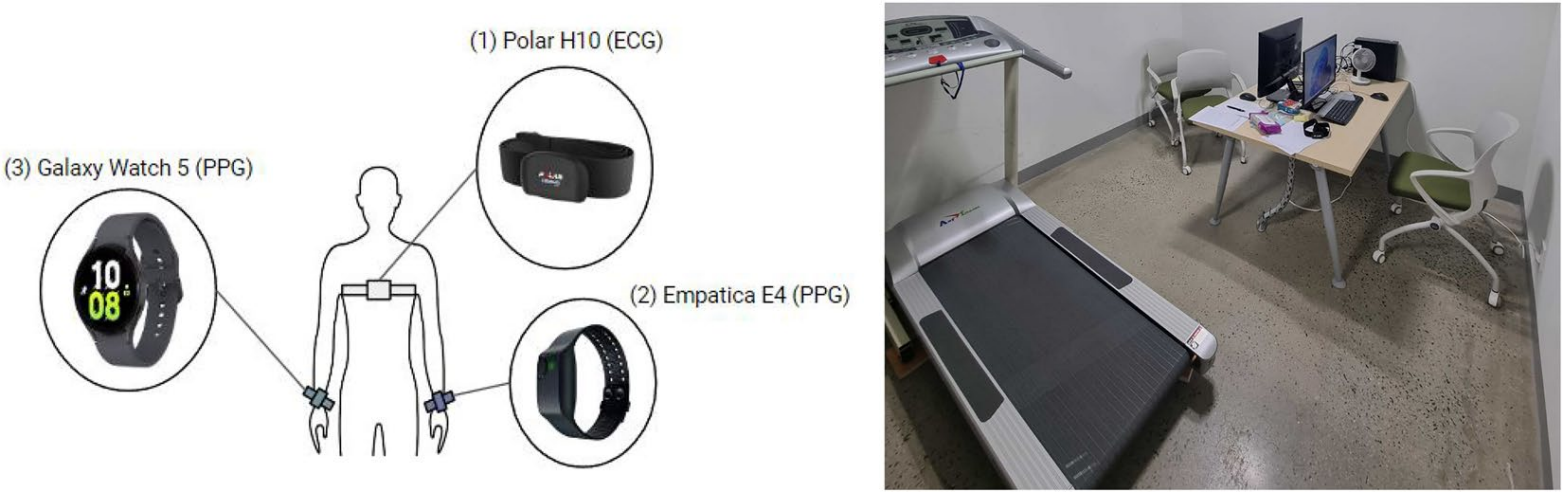
Experimental setup showing the placement of devices on the participant (left) and the laboratory environment (right). Devices attached to the participant: (1) Polar H10 (ECG), (2) Empatica E4 (PPG), and (3) Galaxy Watch 5 (PPG). The positions of the Empatica E4 and Galaxy Watch 5 were randomized for balance. Most activities were conducted at the desk, while walking, jogging, and running were performed on the treadmill.

Participants wore the Polar H10 to measure ECG signals and the Galaxy Watch 5 and Empatica E4 on both wrists to measure PPG signals, as depicted in Fig. 2. Due to physical constraints, wearing both devices on the same wrist was not appropriate. Therefore, the two devices were attached to opposite wrists, and to minimize noise caused by differences in wrist placement, the Galaxy Watch 5 and Empatica E4 positions were counterbalanced by randomizing the placement for each participant. For the Polar H10, a same-gender researcher assisted with fitting the device and ensured it was properly secured before data collection began. Data from the Polar H10 was collected using the Polar Sensor Logger app^8^, and Empatica E4 data was collected using the E4 realtime app^9^.

To replicate daily wear conditions, participants were instructed to wear the Galaxy Watch 5 and Empatica E4 snugly, ensuring a proper fit without causing discomfort. After confirming that all devices were properly worn and operational, participants were given a 5-minute adaptation period, as suggested by Luca *et al*.^10^, to mitigate potential undesired effects caused by tension or unfamiliarity with the equipment. The collection was conducted in the laboratory shown in Fig. 2. A series of tasks requiring monitoring and interaction were performed while participants viewed the screen. To ensure the smooth progress of the data collection, the setup was designed to allow the experimenter to observe the same screen as the participant. Physical activities such as walking and running during the experiment were conducted using a treadmill.

#### Collection Procedure

**Fig. 3:**
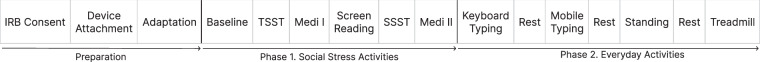
Illustration of the overall data collection protocol, which consists of preparation and two main phases.

The data collection was conducted with two primary objectives, both focused on the PPG signals from the Galaxy Watch: (1) to measure physiological responses to social stress scenarios (as examples of stationary activities) and (2) to assess how much noise (motion artifacts) affects these signals during everyday activities. To achieve both objectives, the collection process was conducted in two consecutive phases: the first phase involved measuring physiological responses in both social stress situations and neutral conditions, followed by the second phase, which focused on diagnosing the effects of various daily movements on PPG signals. The overall procedure followed the steps outlined in Fig. 3.

Before starting data collection, we explained the data collection procedure and obtained IRB consent from the participants. We assisted participants in wearing all three devices and ensured that they were functioning properly before beginning. Additionally, a 5-minute adaptation period was provided to account for initial adjustment before the experiment commenced.

**Phase 1** To induce social stress, the Trier Social Stress Test (TSST)^11^ and the Sing-a-Song Stress Test (SSST)^12^ were adapted and applied. Both tests were selected for their proven effectiveness in reliably inducing social-evaluative stress. The TSST has been widely used in prior work, including the WESAD dataset^3^, to induce stress during physiological signal tracking. Similarly, the SSST has been validated in multiple studies^13,14^ as a practical and effective method for eliciting stress responses, particularly in settings where a simpler or more scalable protocol is desirable. To establish a baseline for physiological responses under neutral conditions, two neutral activities were conducted: baseline^3^ and screen reading^12^. The neutral and stress conditions were conducted sequentially without time intervals, such as prior studies^3,10^. To minimize carryover effects between TSST and SSST, a 5-minute meditation was provided after the stress-inducing tasks to help participants return to a neutral state. To minimize the effects of motion artifacts during this phase, participants were instructed to minimize hand movements as much as possible. However, it is worth noting that participants occasionally moved their hands unconsciously, particularly during the SSST and TSST tasks. The detailed procedure for each activity is described below:

1. *Baseline*: Participants were seated on a chair and allowed to rest for 3 minutes after the adaptation period.
2. *SSST*: During the SSST, stress was induced by asking participants to sing a song. Participants were given 30 seconds to understand the instructions and think about a song they wanted to sing. In the next 30 seconds, they were asked to sing the chosen song. To assess their stress levels, participants were asked to report the degree of stress they experienced immediately after the task using a 7-point Likert scale. Specifically, they were asked: “Please rate your level of stress about the task that just took place using a scale of 1 to 7, where 1 means not at all and 7 means very much.”
3. *Meditation*: After completing the stress-inducing test (i.e., SSST and TSST), a meditation program lasting approximately 5 minutes was provided to help participants return to a neutral state^3^.
4. *Screen Reading*: Participants were instructed to read approximately 15 pre-prepared neutral sentences silently. This activity was conducted for 3 minutes.
5. *TSST*: Participants were asked to imagine themselves in a job interview scenario, following the procedure described by Allen *et al*.^11^. The experimenter acted as the interviewer, asking questions about the participant’s motivation for applying and follow-up questions based on their responses. Participants were given a brief explanation of the scenario and 3 minutes to prepare their answers. The interview itself lasted for three minutes. As in the SSST, participants reported their stress levels on a 7-point Likert scale.

**Phase 2** In the second phase, we aimed to assess the influence of everyday activities on the quality of PPG signals. Everyday scenarios could induce motion artifacts from arm movements, including keyboard and mobile typing, standing, walking, jogging, and running, as shown in previous literature^6,10^. We selected activities to represent different types of motion: wrist movements (i.e., keyboard typing, mobile typing, walking), physical activities (i.e., jogging and running), and postural changes (i.e., standing). Similar to Phase 1, at least a 2-minute break was provided between activities to minimize order effects. At the beginning of the second phase, participants were informed that they no longer needed to restrict their arm movements and could act as they would in their everyday lives. The detailed procedure for each activity is described below:

1. *Keyboard Typing*: Participants typed the text displayed on the screen using a keyboard, and this activity lasted for 3 minutes. We selected and utilized neutral sentences from a Wiki site about watches, following the approach of Brouwer *et al*.^12^.
2. *Mobile Typing*: Participants typed the text displayed on the screen using a smartphone, and this activity also lasted for 3 minutes.
3. *Standing*: Participants were instructed to stand still, and this activity lasted for 3 minutes.
4. *Treadmill*: Participants walked (4-6 km/h), jogged (6-8 km/h), and ran (8-10 km/h) on a treadmill for 2 minutes each, with rest periods provided between activities.
5. *Rest*: Participants were given approximately a 2-minute break between activities to ensure the independence of each activity. For the same reason, adequate rest periods were provided after intensive physical activities (e.g., walking and jogging) to ensure recovery.

## Data Records

**Fig. 4:**
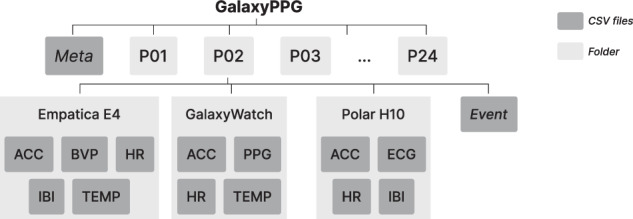
Directory structure for the GalaxyPPG dataset, organized into a hierarchical format. The root directory contains participant information (Meta) and a data folder. Each participant folder (e.g., P01, P02, P24) includes subdirectories for data collected from different devices: Empatica E4, GalaxyWatch, and Polar H10. Each device folder contains corresponding sensor data files (e.g., ACC for accelerometer data, HR for heart rate, etc.).

The GalaxyPPG dataset^15^ is available at Zenodo (10.5281/zenodo.14635823) and organized as shown in Figure 4. At the top level of the directory, there is a file containing metadata about the participants, such as demographic details and experimental conditions. The data for each participant is stored in individual subfolders named according to participant IDs (e.g., P01, P02, up to P24). Within each participant’s folder, the data is further organized by the devices used to collect physiological signals: Empatica E4, Galaxy Watch 5, and Polar H10. A detailed description of each file is provided below:

### Empatica E4

For the E4 data, each record includes timestamps in UTC+0000 with microsecond precision.

- *ACC*: Accelerometer data was collected at 32 Hz. The x, y, and z axes are recorded in 1/64 g-units.
- *BVP*: Blood Volume Pulse data processed from the Empatica E4 at 64 Hz. This data is primarily used to extract HR/HRV features instead of raw PPG data.
- *HR*: Heart rate data calculated by the Empatica E4 at 1 Hz, recorded in beats per minute (bpm).
- *IBI*: Inter-beat interval data calculated by the Empatica E4 with precision in microseconds.
- *TEMP*: Skin temperature data collected from the Empatica E4 at 4 Hz, recorded in degrees Celsius.

### Galaxy Watch

Galaxy Watch data includes timestamps in UTC+0000 with millisecond precision (i.e., timestamp) and when the data was saved due to batching (i.e., dataReceived).

- *ACC*: Accelerometer data was collected at 25 Hz, recording x, y, and z axes. The values were converted to *m*/*s*^2^ using the formula specified in the SDK manual: 9.81/(16383.75/4.0)*value.
- *HR*: Heart rate data calculated by the Galaxy Watch 5 at 1 Hz, recorded in bpm. The status field is also included to denote the status of HR. Further, the IBI data is also included as a list, with error indicators (-1 for error and 0 for normal) accompanying the measurements.
- *PPG*: Raw photoplethysmogram (PPG) data was collected at 25 Hz.
- *SkinTemp*: Skin temperature data collected once per minute, including ambient and body temperatures, recorded in degrees Celsius.

### Polar H10

For Polar H10 data, each record includes timestamps provided by the sensor with nanoseconds precision and the smartphone in UTC+0900 with milliseconds precision.

- *ACC*: Accelerometer data was collected at 200 Hz, with x, y, and z axes recorded in mg.
- *ECG*: Electrocardiogram data collected at 130 Hz, recorded in mV.
- *HR*: Heart rate data was calculated at 1 Hz, recorded in bpm.
- *IBI*: Inter-beat interval data was recorded in millisecond precision.

### Others

- *Meta*: This file contains metadata, including demographic information (e.g., age and gender) and experimental conditions for the participants. The results of the self-reported perceived stress, measured on a 7-point Likert scale, are described in the TSST and SSST fields. The wrist position where participants wore the Galaxy Watch is stated in the “GalaxyWatch” field.
- *Event*: For each participant, there is a log file for the timestamp of activity changes during the experiment. The session field describes the activity, and the status field describes ENTER and EXIT markers.

## Technical Validation

Our technical validation of the dataset consists of the following five components: (1) comparison between GalaxyPPG and existing public datasets, (2) analysis of dataset completeness, (3) evaluation of the physiological validity of PPG signals, (4) analysis of signal differences based on wrist placement, and (5) analysis supporting the dataset’s ecological validity and highlighting its value by demonstrating that activity-induced noise is common in wrist-worn PPG devices.

First, we compared GalaxyPPG with existing public datasets to illustrate how it complements them and contributes to a more comprehensive understanding of PPG signals in real-life contexts. Second, to assess the completeness of the dataset, we analyzed the outlier rate and sampling rate for each sensor modality.

The remaining three analyses focused on deriving physiological metrics from the PPG signals—heart rate (HR), heart rate variability (HRV), and peak matching rate (PMR; the proportion of ECG peaks captured in the PPG signal)—and validating them against ECG-derived ground truth values.

In the third analysis, we evaluated the accuracy of these physiological metrics during sedentary baseline activities to confirm the physiological relevance of the PPG signals. In the fourth, we investigated whether the wrist on which each device was worn affected the measurement outcomes, given that the study design required participants to wear one device on each wrist. In the fifth analysis, we compared physiological metrics extracted from the E4 and Galaxy Watch to show that the observed signal variability reflects realistic noise commonly found in wrist-worn devices. Furthermore, by applying common filtering techniques and demonstrating that such noise cannot be easily removed, we highlight the need for more advanced algorithms in real-world environments and emphasize the value of this dataset for their development and validation.

### Dataset Comparison

We compared the GalaxyPPG dataset with existing datasets that include synchronized PPG and ECG recordings. Three widely used PPG datasets are selected, as summarized in Table 1: IEEE Signal Processing Cup 2015 datasets (IEEE_Training and IEEE_Test)^6^, the WESAD dataset^3^, and PPG-DaLiA^5^. The IEEE datasets serve as foundational benchmarks in PPG heart rate estimation research, while WESAD provides multimodal data for stress and affect detection under controlled laboratory conditions. PPG-DaLiA represents a more recent effort to capture daily activities. This selection enables us to evaluate GalaxyPPG across different scenarios, ranging from traditional controlled experiments to affective computing applications and naturalistic data collection approaches.

**Table 1:** Comparison of PPG Datasets.

Dataset	Activities	Sensor Configuration		Participants
		Modalities	PPG Device	(*n*)
IEEE SP Cup (Training)	Treadmill exercises	ECG PPG ACC	Wrist-worn	12
IEEE SP Cup (Test)	Arm movements Rehabilitation	ECG PPG ACC	Wrist-worn	10
WESAD	3 affective states (neutral, stress, amusement)	ECG PPG ACC TEMP	Empatica E4	15
PPG-DaLiA	8 daily activities (cycling, driving, working, etc.)	ECG PPG ACC TEMP	Empatica E4	15
GalaxyPPG	Stress tests (SSST, TSST) Physical activities Daily tasks (typing, screen reading)	ECG PPG ACC TEMP	Empatica E4 Galaxy Watch 5	24

Notes: ACC: Accelerometer; TEMP: Temperature sensor.

SSST: Sing a Song Stress Test; TSST: Trier Social Stress Test.

The existing datasets differ in their experimental design and scope.

- The IEEE datasets, widely used as benchmarks, employ a device configuration consisting of a single wrist-worn device with dual-channel PPG (515 nm sampling at 125 Hz) and an accelerometer. Their data collection protocols are focused and brief: the IEEE Training dataset includes 12 participants, each performing a 5-minute treadmill exercise session. In contrast, the IEEE Test dataset contains recordings from 10 subjects performing arm movements and rehabilitation exercises.
- WESAD is a multimodal dataset for affective computing research, featuring physiological and motion data collected from 15 participants. Data collection used a carefully designed protocol to generate three distinct affective states: neutral, stress (using the Trier Social Stress Test), and amusement. The sensing backbone of the dataset consists of a chest-worn RespiBAN Professional device that records ECG at 700 Hz, complemented by an Empatica E4 wrist device that captures BVP and acceleration data at varying sampling rates. As in our dataset, WESAD distinguishes itself by integrating stress and emotion detection paradigms, establishing it as a valuable resource for developing affect recognition systems.
- PPG-DaLiA represents a more recent approach that focuses on capturing activities of daily life. Using Empatica E4 and RespiBAN devices for BVP and ECG recordings, this dataset includes 15 subjects performing various activities under semi-controlled conditions. Although it mainly analyzes motion artifacts, its scope includes eight daily activities such as cycling, driving, and working.
- GalaxyPPG builds upon and extends previous approaches in several key aspects. First, it employs a comprehensive three-device configuration: the commercial device Galaxy Watch 5 (providing PPG at 25 Hz, accelerometer data at 25 Hz, and temperature data at 1/60 Hz), the Empatica E4 (capturing BVP at 64 Hz, accelerometer data at 32 Hz, and temperature data at 4 Hz), and the Polar H10 (recording ECG at 130 Hz as ground truth). In contrast to earlier datasets focusing exclusively on physical tasks (e.g. IEEE) or controlled stress conditions (e.g., WESAD), GalaxyPPG broadens the experimental scope by incorporating both physical activities and psychological stress tests, along with the natural transitions between them. This dataset includes data from 24 participants. In comparison, the WESAD and PPG-DaLiA datasets each contain data from 15 participants, and the IEEE Training and Test datasets contain data from 12 and 10 participants, respectively.

**Heart rate distributions** To quantitatively compare these datasets, we analyzed their heart rate distributions, as summarized in Table 2. We segmented the heart rate ranges into 20 bpm intervals from 0 to 200 bpm to reveal distribution patterns across different activity types. This segmentation allows us to examine how heart rates cluster during various daily activities; for instance, the 60–80 bpm range typically corresponds to light activities like sitting or walking, whereas ranges above 120 bpm generally reflect more intense physical exertion^16^. This analysis is essential for understanding the physiological range and variability captured by each dataset, highlighting how the distribution patterns align with each dataset’s intended purpose. Notably, GalaxyPPG, WESAD, and PPG-DaLiA contain significantly more samples (35,691; 22,478; and 64,697, respectively) than the IEEE datasets (1,768 and 1,328), providing a richer data pool for analysis.

**Table 2:** Heart Rate Distribution and Statistics Across Different Datasets.

Statistics	GalaxyPPG	WESAD	PPG-DaLiA	IEEE-D1	IEEE-D2
Total Samples	35,691	22,478	64,697	1,768	1,328
HR Range: 0–40 bpm	0	0	0	0	0
HR Range: 40–60 bpm	203	2,440	3,746	0	6
HR Range: 60–80 bpm	16,739	12,688	21,585	59	235
HR Range: 80–100 bpm	13,315	5,062	22,374	118	240
HR Range: 100–120 bpm	2,689	1,275	9,884	248	153
HR Range: 120–140 bpm	1,094	905	4,878	356	96
HR Range: 140–160 bpm	1,040	104	1,679	696	247
HR Range: 160–180 bpm	557	0	512	256	350
HR Range: 180–200 bpm	54	0	39	0	0
Average HR (bpm)	86.70	78.07	89.43	135.95	115.39
Standard Deviation of HR (bpm)	20.98	13.49	22.83	24.30	31.08

Note: All datasets were analyzed using an 8-second sliding window with a 2-second step size.

For the WESAD dataset, HR values were extracted from ECG signals during activities (baseline, stress, amusement, and meditation conditions). Undefined and ignored labels were excluded.

Importantly, the heart rate distribution in GalaxyPPG more accurately represents real-world scenarios, with 84.2% of measurements falling within the 60–100 bpm range, typically associated with everyday activities. In contrast, the IEEE datasets skew toward higher heart rates (average 135.95 and 115.39 bpm), reflecting their focus on intensive physical exercises such as treadmill running. WESAD’s narrower heart rate range (predominantly between 60–120 bpm) is particularly well-suited to its focus on affect and stress detection in controlled laboratory settings, whereas GalaxyPPG’s wider distribution better supports its aim of monitoring naturalistic daily variations. While GalaxyPPG and PPG-DaLiA exhibit similar heart rate distributions (mean HR of 86.70 and 89.43 bpm, respectively), GalaxyPPG is unique in its inclusion of psychological stress tests (TSST and SSST) and the incorporation of a consumer-grade wearable device (the Galaxy Watch) alongside research-grade sensors.

**Fig. 5:**
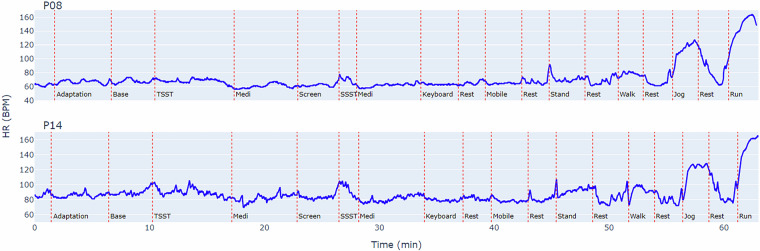
Comparison of heart rate variations across different activities for two participants (P08 and P14).

To illustrate the dynamic heart rate trends captured in our dataset, Fig. 5 presents the heart rate time series for participants P08 and P14 from the GalaxyPPG dataset. This figure visually demonstrates the dataset’s capability to capture a wide range of physiological responses, from high-intensity physical challenges like running (140–160 bpm) to the more subtle variations induced by psychological stressors during TSST and SSST (60–100 bpm). Furthermore, it highlights the individual differences in cardiovascular responses to similar stimuli, underscoring the dataset’s rich potential for personalized health monitoring research.

### Dataset Completeness Analysis

To establish the dataset’s reliability and usability, we systematically assessed data completeness across all sensor modalities.

Note that it is important to acknowledge a data collection misconfiguration that affected participant P01: their Galaxy Watch data and corresponding event annotations were not recorded due to an error in the device configuration setup. While we have retained this participant’s other sensor data (from the Empatica E4 and Polar H10) for potential alternative uses, these records were excluded from the technical validation analyses presented in this paper. Additionally, we observed partial data loss from the Galaxy Watch during otherwise complete recording sessions: heart rate measurements were missing for participants P07 and P08, and skin temperature data was absent for P02. These gaps were included in our missing rate calculations to accurately reflect real-world usage and data variability.

For the remaining recordings, we calculated the outlier rate for each sensor modality. Following the methodology proposed by Sukor *et al*.^17^, we defined a weak signal criterion based on the peak-to-peak amplitude relative to signal variance for PPG and ECG signals:

1 $$\,{\rm{WeakSignal}}\,(x)=\left\{\begin{array}{ll}1, & \,{\rm{if}}\,\max (x)-\min (x) < 0.1\,{\sigma }_{x},\\ 0, & \,{\rm{otherwise}}\,.\end{array}\right.$$

where *x* represents the non-NaN signal values and *σ*_*x*_ is the signal’s standard deviation.

For accelerometer data, we identified invalid measurements by checking for values exceeding 10 g, as the work by Lee *et al*.^18^ has shown that typical daily activities generally remain within 0.5 g to 10 g. Heart rate measurements were considered valid if they fell within the physiologically reasonable 40–200 bpm range, following the criteria established by Bashar *et al*.^19^ We also incorporated the Galaxy Watch’s internal status codes, officially provided by the SDK and collected through our custom data collection tool, to identify periods when the device itself deemed the heart rate calculation unreliable.

As shown in Table 3, most of the sensor modalities demonstrated high data quality with low missing rates. The PPG, BVP, and ECG signals demonstrated high reliability with low missing rates, validating their use. However, the HR data from the Galaxy Watch showed a notably higher missing rate of 21.43% as heart rate measurements were missing for participants P07 and P08.

**Table 3:** Missing rate (%) of each signal.

Galaxy Watch 5			Empatica E4			Polar H10		
PPG	ACC	HR	BVP	ACC	HR	ECG	ACC	HR
0.14	0.04	21.43	0.01	2.93	0.00	0.62	0.56	0.06

Data quality was further verified by analyzing the actual sampling rates achieved by each device. Table 4 presents the measured sampling rates across the three wearable devices (E4, Galaxy Watch, and Polar H10) for various physiological and motion signals. The sampling rate and its standard deviation were calculated based on the time intervals between consecutive data points, using the complete set of samples from all participants.

**Table 4:** Cross-device Sampling Rate Analysis.

Device	Signal	Expected (Hz)	Measured (Hz)	Total Samples
Galaxy	PPG	25.00	24.90 ± 0.06	1,851,318
	ACC	25.00	24.90 ± 0.06	1,851,318
	HR	1.00	1.00 ± 0.01	67,810
	TEMP	0.02	0.02 ± 0.00	1,085
E4	BVP	64.00	64.00 ± 0.01	4,759,074
	ACC	32.00	32.00 ± 0.01	2,379,533
	HR	1.00	1.00 ± 0.00	74,365
	TEMP	4.00	4.00 ± 0.00	297,437
Polar	ECG	130.00	130.49 ± 0.10	9,703,051
	ACC	200.00	202.37 ± 0.95	15,048,539
	HR	1.00	1.00 ± 0.01	74,361

Analysis shows that most signals were recorded near their specified rates, indicating generally reliable data acquisition. However, we observed some deviations between nominal and actual sampling rates. These discrepancies are consistent with findings from previous studies of wearable sensor performance in real-world conditions, where factors such as processing overhead and power management can affect sampling behavior^20^.

Our data completeness analysis demonstrates that the dataset was reliably collected, with most sensor modalities showing low missing rates and sampling rates consistently matching device specifications.

### Signal Processing

**Fig. 6:**
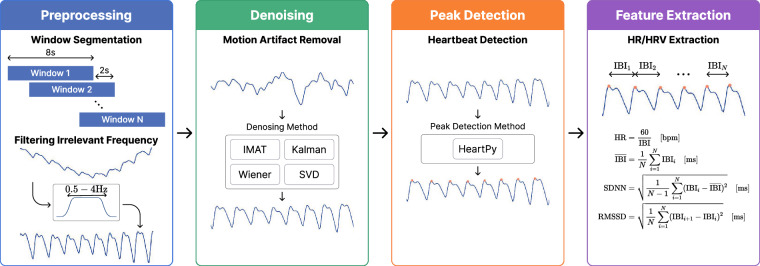
Overview of the PPG signal processing pipeline. The pipeline consists of four main stages: (1) preprocessing with window segmentation and bandpass filtering, (2) motion artifact reduction using four different denoising approaches (IMAT, Kalman, Wiener, and SVD), (3) peak detection using the HeartPy framework, and (4) HR/HRV measurement calculation. Each stage is designed to improve signal quality progressively and extract meaningful cardiovascular parameters.

The comparative analysis of heart rate (HR) and heart rate variability (HRV) derived from PPG and ECG signals plays a key role in establishing the technical validity of this dataset. Prior to presenting the results, we briefly outline the methods used to compute HR and HRV, as summarized in Fig. 6.

#### Preprocessing

PPG signals were preprocessed using a two-stage approach (Fig. 6). First, we applied temporal segmentation using 8-second windows with 2-second overlaps between consecutive windows. The selection of an 8-second window duration has been a common choice in the literature, as demonstrated in prior studies^21–24^. Furthermore, Baek *et al*.^25^ provided empirical evidence supporting the reliability of HRV calculations even within relatively short time windows, validating our choice of an 8-second duration for both HR and HRV analysis. For the second stage, we applied specific filtering techniques tailored to each device’s characteristics. The Galaxy Watch PPG signals underwent bandpass filtering within the 0.5–4 Hz frequency range, a crucial step for mitigating baseline wander artifacts^4^. The Empatica E4’s BVP signals may not require additional filtering due to their internal preprocessing algorithms^26^. However, the same bandpass filter was also applied to the E4’s BVP signals for comparison with the Galaxy Watch PPG signals.

#### Denoising

Various algorithms have been developed to address *motion artifacts* in PPG signals, utilizing diverse signal processing techniques. While modern techniques often combine multiple principles for better performance^27^, we can identify four primary theoretical foundations that form the basis of most methods: signal decomposition, iterative reconstruction, adaptive filtering, and statistical estimation. Although many successful algorithms integrate multiple processing techniques, we classify them based on their principal framework to facilitate systematic comparison.

**Signal decomposition** methods break down PPG signals into separate components to isolate motion artifacts from the underlying physiological signal. The Singular Value Decomposition (SVD) technique presented in Reddy *et al*.^28^ exemplifies this approach, using matrix factorization to separate the original signal into distinct components that can be analyzed independently. TROIKA^6^ builds on this concept by employing Singular Spectrum Analysis (SSA) to decompose the signal, followed by sparse reconstruction to rebuild the cleaned signal.

**Iterative reconstruction** approaches, exemplified by the Iterative Method with Adaptive Thresholding (IMAT)^29^, primarily leverage signal sparsity through multiple reconstruction iterations. In each iteration, the algorithm applies increasingly precise thresholds to separate the true signal from noise, using spectral analysis to guide this refinement process.

**Adaptive filtering** methods continuously adjust their parameters based on changing signal conditions. The Kalman filtering approach^30^ implements this concept by maintaining a dynamic model of both the PPG signal and motion artifacts, continuously updating its estimates based on new measurements. Similarly, SpaMA^22^ primarily employs adaptive spectral analysis while incorporating elements of signal decomposition to identify and remove motion-related components based on accelerometer data.

**Statistical estimation** techniques focus on spectral characteristics of signals and noise. The Wiener filter with Phase Vocoder (WFPV)^31^ exemplifies this approach, combining minimum mean square error filtering in the frequency domain with phase-based frequency refinement. While primarily operating in the frequency domain, WFPV incorporates adaptive elements to handle time-varying motion artifacts.

These methods (SVD for signal decomposition, IMAT for iterative reconstruction, Kalman filtering for adaptive estimation, and Wiener filtering for frequency-domain processing) were selected to evaluate the dataset’s technical validity, representing diverse approaches to artifact removal. While each method may incorporate complementary techniques, this selection provides a comprehensive evaluation. To ensure a fair comparison focused on the denoising capabilities relevant to HRV analysis, we implemented only the denoising components of each algorithm. This was necessary because the original peak detection components were designed for frequency-domain HR estimation rather than the time-domain peak detection required for HRV. By using only the denoising components, we could apply a standardized peak detection method across all denoised signals, enabling a systematic comparison of temporal characteristics crucial for HRV measurement.

#### Peak Detection

**Fig. 7:**
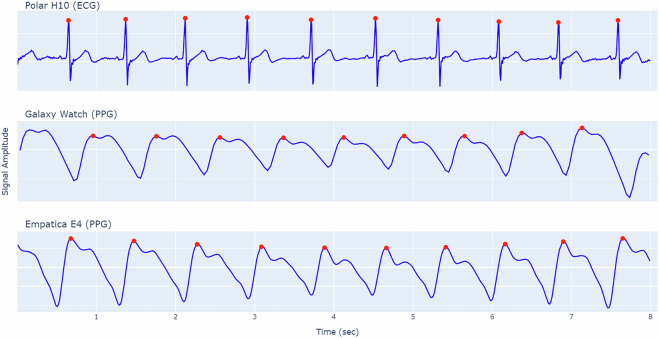
Illustrative examples of ECG and PPG signals collected from each device during baseline activity, including the detected peaks based on data from P02.

We employed HeartPy^32,33^ for ECG and PPG calculation in the validation process, an open-source framework that has become a popular tool in physiological signal processing research^34–36^. HeartPy implements a time-domain approach that combines adaptive thresholding with moving averages, enabling the detection of cardiac events even in noisy conditions. The framework is suitable for extracting both R peaks from ECG signals and systolic peaks from PPG signals, as illustrated in Fig. 7. A post-processing function inspired by Allen’s methodology^37^ was implemented to validate peaks through adaptive thresholding and interpolate potential missed beats. This peak detection capability, validated extensively on real-world physiological data, allows for reliable calculation of various HRV metrics from IBI.

ECG signal analysis accuracy depends on the filtering parameters used during preprocessing, as demonstrated by Altay *et al*.^38^. This relationship between filtering frequency bands and QRS detection has been extensively investigated by Elgendi *et al*.^39^, who showed that a broader 5–40 Hz bandwidth effectively preserves QRS complex morphology. While Sadhukhan *et al*.^40^ established that optimal R-peak detection typically occurs within a narrower 5–15 Hz range, we found that applying this single fixed band could limit detection accuracy across our diverse dataset segments. Therefore, an adaptive filtering approach was implemented, evaluating multiple bandpass configurations (5–15 Hz to 5–40 Hz in 5 Hz increments) for each 8-second window to optimize HeartPy’s peak detection performance while maintaining physiological validity. Filter settings for each window have been documented in the code for transparency and reproducibility.

To assess how well the systolic peaks in PPG signals (from E4 and Galaxy Watch) correspond to the reference ECG R-peaks (from Polar H10), we evaluated the correspondence between ECG R-peaks (ground truth from Polar H10) and PPG systolic peaks (from E4 and Galaxy Watch). We identified the closest ECG R-peak for each PPG systolic peak within a 0.5-second window. This window width was chosen based on typical Pulse Transit Time (PTT) values in healthy individuals, as PTT rarely exceeds 500 ms from the R-wave to peripheral pulse arrival^41^. Once a PPG peak is matched to an ECG R-peak, it becomes unavailable for subsequent matching, ensuring each peak is matched at most once. This approach accounts for the physiological Pulse Transit Time (PTT)^42^ between ECG R-peaks and PPG systolic peaks while maintaining the temporal sequence of cardiac events. Detection accuracy was quantified using the Peak Matching Rate (PMR), calculated as the ratio of matched ECG peaks to the total number of ECG peaks per window.

#### Feature Extraction

We focused our analysis on two key cardiovascular parameters: HR and HRV. These metrics were chosen for their complementary nature in assessing cardiovascular function—while HR provides insight into overall cardiac activity, HRV offers a more nuanced view of autonomic nervous system regulation^43^.

Several HRV metrics reflecting different aspects of cardiac function were analyzed. The mean inter-beat interval (IBI) measures the average time between consecutive heartbeats, providing insight into overall heart rhythm. The Standard Deviation of NN intervals (SDNN) quantifies heart rate variability by measuring the variation between successive normal heartbeats. “NN intervals” represent the time between consecutive normal QRS complexes in the electrocardiogram, excluding abnormal beats. The Root Mean Square of Successive Differences (RMSSD) captures beat-to-beat variations and is particularly sensitive to short-term heart rate changes, making it valuable for assessing autonomic nervous system function. These metrics have demonstrated significant utility across various health monitoring applications, from evaluating cardiovascular health to assessing mental stress responses^44–46^.

### Evaluation of Physiological Validity of PPG Signals

Physiological metrics were extracted from the PPG signals collected during the baseline activity (in which participants remained seated with minimal movement) using the signal processing framework without applying denoising. These metrics were then compared with ECG-derived metrics to assess the physiological validity of the collected PPG signals.

As shown in Table 5, heart rate measurements during the baseline period were highly consistent across all devices: 75.89 bpm from the Galaxy Watch, 78.64 bpm from the E4, and 78.47 bpm from the Polar H10 reference. This consistency indicates the collected PPG signals are physiologically valid.

**Table 5:** Comparison Across Activities and Devices Using HeartPy Algorithms without Denoising. (Metrics: HR in bpm, IBI/SDNN/RMSSD in ms, PMR in %).

Activity	Polar H10 (ECG)				Galaxy Watch (PPG)					Empatica E4 (PPG)				
	HR	IBI	SDNN	RMSSD	HR	IBI	SDNN	RMSSD	PMR	HR	IBI	SDNN	RMSSD	PMR
Baseline	78.47	775.09	40.86	41.63	75.89	810.40	136.25	185.79	92.08	78.64	781.07	103.83	137.15	96.03

### Impact of Wrist Placement on Signal Accuracy

To evaluate the impact of wrist placement on PPG signal accuracy, we conducted a paired *t*-test using the MAE of HR derived from simultaneously recorded signals from both devices. This approach naturally controls for inter-individual variability by comparing left and right wrist data from the same participant. Furthermore, the counterbalanced experimental design minimized the influence of device-specific differences, allowing the effect of wrist placement to be assessed more accurately. The analysis revealed no significant difference between the left and right wrists (Left: 15.88 ± 4.75 bpm; Right: 15.26 ± 5.30 bpm; *t*(22) = 0.459, *p* = 0.651). This result suggests that wrist placement did not significantly affect the accuracy of HR measurement under the experimental conditions, thereby supporting the validity of the collection setup.

### Analysis of Activities to Support Ecological Validity

Heart rate (HR) and heart rate variability (HRV) values were calculated from the PPG signals collected during each activity. Denoising techniques, including IMAT, Kalman, SVD, and Wiener filters, were applied, and the HR and HRV values obtained using the Wiener filter, which showed the greatest overall improvement in accuracy, are summarized in Table 6.

**Table 6:** Comparison Across Activities and Devices Using HeartPy Algorithms with Wiener Denoising.

Activity	Polar H10				Galaxy Watch					E4				
	HR	IBI	SDNN	RMSSD	HR	IBI	SDNN	RMSSD	PMR	HR	IBI	SDNN	RMSSD	PMR
Baseline	78.47	775.09	40.86	41.63	78.15	778.78	55.22	71.78	95.36	79.34	769.14	67.34	83.89	95.86
TSST	88.15	690.68	48.38	47.55	84.54	718.74	86.18	116.91	91.72	87.02	698.89	72.81	97.64	94.36
Screen Reading	79.17	769.01	37.58	38.81	79.09	770.27	57.38	75.34	94.87	82.26	744.55	67.33	84.78	95.85
SSST	94.64	647.14	42.64	44.42	89.50	685.51	93.85	129.55	90.68	92.38	662.09	77.87	102.16	94.19
Keyboard Typing	78.69	770.11	30.12	33.80	79.29	763.84	128.38	178.68	92.88	82.78	732.05	121.74	166.86	94.95
Mobile Typing	76.95	786.31	34.51	39.30	76.91	787.31	65.56	91.58	95.08	77.69	780.61	74.61	101.90	95.59
Standing	88.47	689.74	36.29	34.33	86.58	704.85	71.37	96.74	93.71	88.20	691.47	65.70	86.01	94.78
Walking	100.72	601.97	28.55	33.88	97.90	622.95	127.57	173.24	91.43	108.26	570.98	112.63	152.28	94.53
Jogging	133.76	457.00	45.80	60.98	98.05	641.19	145.71	194.88	72.95	97.88	643.80	135.18	179.12	72.67
Running	154.19	394.36	51.60	69.18	95.33	656.04	119.49	159.01	62.30	99.29	628.80	127.56	165.27	64.87

Metrics: HR in bpm, IBI/SDNN/RMSSD in ms, PMR in %.

However, even with filtering applied, activities involving moderate physical movement, such as jogging, still showed significant discrepancies in heart rate compared to ECG (Galaxy Watch: 98.29 bpm, E4: 97.88 bpm, H10: 133.76 bpm). In HRV measurements, both the Galaxy Watch and E4 exhibited substantial differences from the ECG across all activities, with particularly large discrepancies in the SDNN and RMSSD values, though the E4 showed somewhat better alignment in these metrics.

In summary, each device maintained a consistent sampling rate with a precision within 2% of the specified specifications, supporting the dataset’s completeness. A comparison between PPG from the Galaxy Watch and ECG during the baseline activity showed low error rates (3.80% for HR and 4.36% for IBI) with 92.08% of ECG-derived beats accurately detected in the PPG signal, confirming that PPG signal reflect physiologically valid. In addition, we confirmed that there was no significant effect of the wrist on which the wearable was worn.

Lastly, discrepancies between the PPG data from the Galaxy Watch and ECG were observed, but similar differences were also noted with the E4 device, even after noise filtering. Considering that accurate measurements were obtained at baseline, this suggests that the discrepancies are not due to issues with the dataset collection setup, but rather motion artifacts commonly encountered with wrist-based PPG devices. Therefore, these observations do not represent a limitation of the dataset’s technical validity, but rather support the motivation for presenting this dataset, which aims to provide realistic physiological signals that reflect real-world conditions.

## Usage Notes

This dataset was collected using a commercial wearable device, the Samsung Galaxy Watch, and includes PPG signals recorded during various activities. It is potentially helpful for research on motion artifacts in PPG signals. However, several limitations should be considered when utilizing the data. At the time of data collection, the Samsung Health Sensor SDK supported only single-channel PPG (green wavelength). As such, the dataset contains only green-channel PPG data. Although recent SDK updates now enable multi-wavelength data collection, including infrared (IR) and red channels, this dataset does not include such multi-channel data. The dataset includes a variety of activity scenarios to reflect everyday daily movements, but it does not cover the full spectrum of real-life activities. For broader generalization, additional data collection in naturalistic settings, along with detailed activity tracking, may be necessary. It is also important to note that the dataset was collected in South Korea, and all participants were of East Asian descent. While exact skin color information was not explicitly recorded, participants are likely to correspond to Fitzpatrick skin types III-IV, which are typical for East Asian populations. Additionally, when using the dataset, please note that the PPG signals from the Galaxy Watch are provided as raw signals and must be inverted prior to analysis, as the device uses a reflective-type PPG sensor.

## Data Availability

We developed data collection applications for the Galaxy Watch and used them to compile the GalaxyPPG dataset, available at https://github.com/Kaist-ICLab/GalaxyPPG-Logger. The repository contains the version of the logger application used for the GalaxyPPG data collection to ensure scientific rigor. For future use, we are actively maintaining and updating the logger application. Please refer to the GitHub repository for detailed information. Additionally, technical validity assessments and data exploration were performed using Python scripts, which are available at https://github.com/Kaist-ICLab/GalaxyPPG-Supplementary-Code.
